# 1295. Cross-sectional survey on education of clinical infectious diseases and antimicrobial resistance in pharmaceutical department in Japanese universities

**DOI:** 10.1093/ofid/ofac492.1126

**Published:** 2022-12-15

**Authors:** Ryuji Koizumi, Chika Tanaka, Mio Endo, Yumiko Fujitomo, Shinya Tsuzuki, Yusuke Asai, Akane Ono, Yuichi Muraki, Takashi Kitahara, Norio Ohmagari

**Affiliations:** National Center for Global health and Medicine, Shinjuku, Tokyo, Japan; National Center for Global health and Medicine, Shinjuku, Tokyo, Japan; National Center for Global health and Medicine, Shinjuku, Tokyo, Japan; National Center for Global health and Medicine, Shinjuku, Tokyo, Japan; National Center for Global Health and Medicine, Shinjuku-ku, Tokyo, Japan; National Center for Global Health and Medicine, Shinjuku-ku, Tokyo, Japan; National Center for Global health and Medicine, Shinjuku, Tokyo, Japan; Kyoto Pharmaceutical University, Kyoto, Kyoto, Japan; Yamaguchi University Hospital, ube, Yamaguchi, Japan; National Center for Global Health and Medicine, Shinjuku-ku, Tokyo, Japan

## Abstract

**Background:**

Pharmacists play an important role in infection control and antimicrobial stewardship teams in hospitals and the community. The need for education on antimicrobial resistance (AMR) and clinical infectious diseases (CID) is increasing. However, the status of Japanese pharmaceutical education on CID and AMR is unclear.

**Methods:**

We conducted a nationwide cross-sectional survey of pharmaceutical department in Japanese universities between February and March 2022. Data on the following were collected using questionnaires: 1) basic information; 2) number of faculty members with experience in CID; 3) lecture times for CID and AMR; 4) self-evaluation of CID and AMR education; and 5) the most important issues in CID and AMR education. Descriptive analyses were conducted.

**Results:**

Among the 74 participating universities, 44 (59.5%) universities responded. The median number [IQR] of faculties of infectious disease education was 7 [4–12], and clinical faculties was 3 [1–6]. Twenty-seven (62.8%) of the universities had faculties with clinical experience in infectious diseases. Lectures time for CID and AMR varied among the universities.

Respondents indicated that CID and AMR lectures were insufficient, compared to microbiology basic lectures. Additionally, insufficient lecture time and specialists were the most common responses as the most important issues in CID and AMR education.

University background and questions regarding CID and AMR lectures (N=44)

**Figure:**
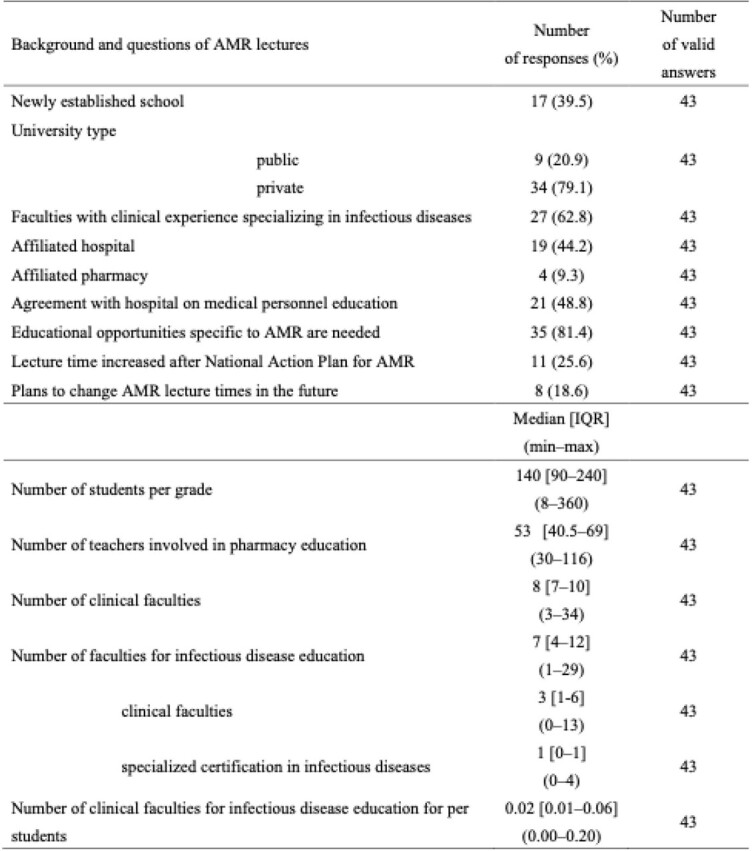

Boxplot of lectures time for clinical infectious disease and antimicrobial resistance education

**Figure:**
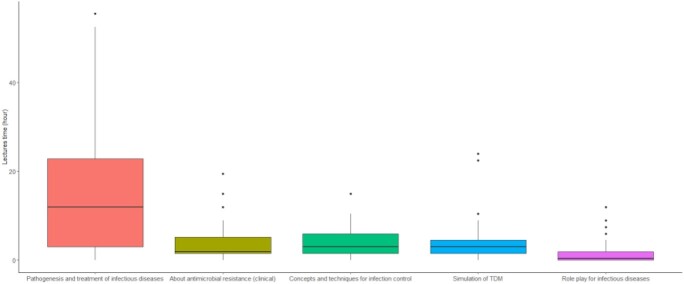

Self-evaluation of clinical infectious disease and antimicrobial resistance education

**Figure:**
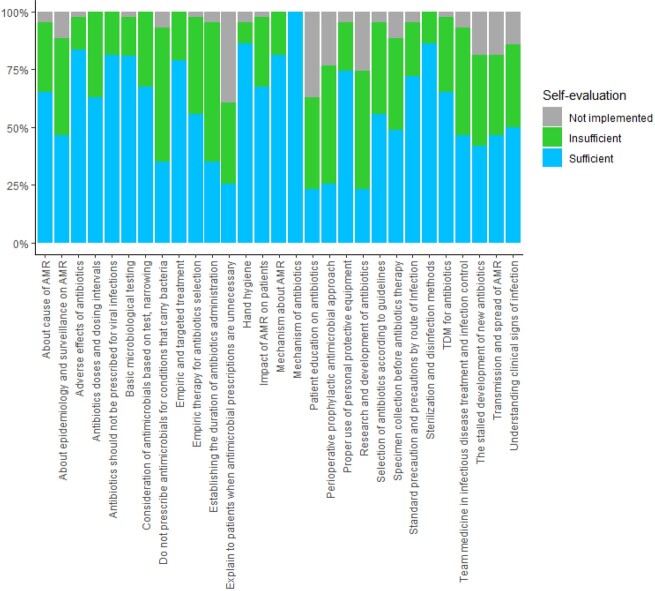

**Conclusion:**

This study revealed that educational status and resources for CID and AMR education varied widely, and that practical lectures on CID and AMR may be insufficient. We suggest that the entire core curriculum and resources, including the number of faculties, needs to be examined and improved.

The most important issues in clinical infectious disease and antimicrobial resistance education.

**Figure:**
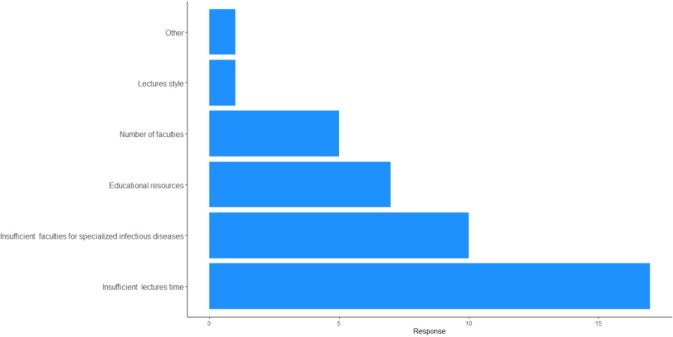

**Disclosures:**

**All Authors**: No reported disclosures.

